# Dietary supplementation of *Bacillus*-based probiotics on the growth performance, gut morphology, intestinal microbiota and immune response in low biosecurity broiler chickens

**DOI:** 10.1016/j.vas.2021.100216

**Published:** 2021-11-04

**Authors:** Mohammad Arif, Md. Akteruzzaman, Tuhin-Al-Ferdous, Sk Shaheenur Islam, Bidhan Chandra Das, Mahbubul Pratik Siddique, S. M. Lutful Kabir

**Affiliations:** a Department of Microbiology and Hygiene, Bangladesh Agricultural University, Mymensingh-2202, Bangladesh.; b Department of Livestock Services, Krishi Khamar Sarak, Farmgate, Dhaka 1215, Bangladesh

**Keywords:** Bacillus licheniformis, Bacillus subtilis, broiler chicken, probiotic, gut histomorphology, intestinal microflora, immune response, low biosecurity

## Abstract

A feeding trial was conducted to evaluate the effects of *Bacillus*-based probiotics on growth performance, intestinal histo-morphology, gut microbial population and immune response in broilers. A total of 2000 Hubbard Classic day-old chicks were randomly enrolled in four experimental groups and 4 replicates of 500 birds in each group, and reared for 35 days under a low- level of biosecurity measures. The trial groups were assigned treatment-1 (T1): basal diet(control), treatment-2 (T2): basal diet plus *Bacillus licheniformis* (DSM17236), treatment-3 (T3): basal diet plus *Bacillus subtilis* (PB6), and treatment-4 (T4) basal diet plus 4% Flavomycin. All four groups were fed with maize-soybean based prepared feeds (starter, grower and finisher). Dietary inclusion of *B. licheniformis* significantly improved body weight gain and lessened FCR in T2 compared to other groups (*p <* 0.05). Probiotics increased the population of *Bacillus* spp. and decreased the population of *Clostrium perfringens, Salmonella* spp. and *Escherichia coli* in the jejunum and ileum in broiler birds on day 21 and 35 (*p <* 0.05). The highest antibody production was observed in *B. licheniformis* treated group (T2) compared to other probiotic treated group (T1). Taken together, the study findings suggest that *B. licheniformis* probiotics could be used as a feasible alternative to antimicrobials in the broiler production considering beneficial impacts at low biosecurity broiler farms.

## 1 Introduction

Antimicrobial agents have been used in commercial poultry production as feed supplements more than 50 years ago because of feed efficiency and growth promotion via modulation of intestinal microflora, and disease prevention as the effect of improved host immunity (Bunyan, Jeffries, Sayers, Gulliver, & Coleman, 1977). However, imprudent antimicrobial use (AMU) in the poultry production have developed antimicrobial residues including the emergence of antimicrobial resistant (AMR) microbes, which could lead to great risk to public health (Shivaramaiah et al., 2011). In addition, intensive poultry rearing causes stress in birds, resulting reduction of immune response. This phenomenon significantly enhances intestinal colonization of pathogens (O'Dea et al., 2006). Therefore, alternative options are needed that act similar to antibiotics targeted for control of disease producing microorganisms, and support to the growth performance of poultry. Thus, replacement of prohibited antibiotic growth promoters (AGP) has grew more attention during the recent past (Deniz et al., 2011). Probiotics as a live microbial feed supplement provide beneficial effects on the host through enhancing intestinal microbial balance (Fuller, 1989). At present, a number of microbial species have been used as probiotics, including species of *Bacillus, Bifidobacterium, Enterococcus, Lactobacillus, Lactococcus, Streptococcus, Pediococcus, Aspergillus, Candida,* and *Saccharomyces* etc. (O'Dea et al., 2006), of which species under the *Lactobacillus* and *Bifidobacterium* are widely used (Park et al., 2021). Notwithstanding, years of experience for safe commercial use of *Bacillus* spp. including *B. subtilis, B. licheniformis* and *B. coagulans* as potent probiotics in animal production has increased, but their application has been shadowed by the use of these two dominant genera both in human and animal health (AlGburi et al., 2016; Mahoney et al., 2019). In different ways, probiotics can work in the gastrointestinal tract (GIT) of poultry through competitive exclusion and antagonism process (Kabir et al., 2005; Kizerwetter-Swida & Binek, 2009), modification metabolism through intensifying digestive enzyme activity, and reducing ammonia production including bacterial enzyme activity (Yoon et al., 2004), improving feed intake, digestion and absorption capacity (Awad, Böhm, Razzazi-Fazeli, Ghareeb, & Zentek, 2006), and boost body immune response (Apata, 2008; Mathivanan & Kalaiarasi K., 2007). However, major benefits of using probiotics in livestock and poultry production to prevent disease, and growth improvement by enhancing feed efficiency and reducing mortality rate as well have been observed (Deniz et al., 2011).

Enteric diseases are considered to be immense burden to the poultry industry as they are associated with decreased weight gain, higher FCR, increased mortality rate, higher medication costs, and increased likelihood of contamination in poultry products with zoonotic pathogens for human infection (Timbermont, Haesebrouck, Ducatelle, & Van Immerseel, 2011). Intestinal diseases are associated with the overgrowth of *Clostridium perfringens, Salmonella* spp., and even *Escherichia coli* in the GIT of poultry. Necrotic enteritis (NE) frequently occurs in poultry, which is caused by *Clostridium perfringens* and typically occurs in broiler chickens between 2 to 6 weeks of age. *Bacillus licheniformis* strain of probiotics would prevent NE and to improve growth in broiler poultry (Cheng et al., 2017). Other important bacterial pathogens like *Salmonella* spp. (Shivaramaiah et al., 2011) and *E. coli* (Cooper, Songer, & Uzal, 2013), which may cause enormous economic loss to the poultry industry through diminishing overall performance and increasing mortality rate in poultry. All these persuade a very high economic burden to the farmers including public health risks.

The use of *Bacillus* based probiotics (*B. licheniformis* and *B. subtilis*) could be potential AGP replacements in the broiler feed considering the emergence of AMR, and support to the cost-effective poultry production in low resource settings like Bangladesh. Therefore, this study was conducted to evaluate influence of *Bacillus-* based probiotics in growth performance, intestinal histo-morphology, gut microbial population and immune response status in low biosecurity broiler flocks in comparison with AGP. The findings of this study will promote to scale up this practice in the majority broiler production systems in Bangladesh where lower standard of biosecurity measures is most common.

## 2 Materials and Methods

### 2.1 Experimental design, diet and bird management

Two thousand (2000) day-old chicks of commercial Hubbard Classic broiler with an average initial body weight of 45.2 ± 0.2 g were collected from a local chick supplier and randomly assigned into 4 dietary treatment groups which included 500 birds in each group with 4 replicates (125 birds/replicate). The birds were kept under low level of biosecurity measurements of the sector three poultry production systems of FAO classification (Dolberg, 2008). The potential characteristics of low biosecurity parameters were as inadequate provision of perimeter fencing and netting of the poultry farm/shed, quarantine facilities, footbath, separate boot and clothing of the poultry workers for farm use, poultry waste management including cleaning and disinfection practices (FAO, 2008; Akther et al., 2018).

The experimental trial was conducted for a 35 day-period and the birds were fed with a prepared poultry ration (starter, grower and finisher) and divided into four groups based on desired combination: (1) treatment-1/control (T1): birds were fed as a basal diet only; (2) treatment-2 (T2): birds were fed a basal diet plus *Bacillus licheniformis* DSM17236 (Gallipro Tect®, Chr. Hansen Holding A/S, Denmark, 3 × 10^9^CFU/g), 1.0 Kg/metric ton (MT) feed; (3)treatment-3 (T3): birds fed a basal diet plus *Bacillus subtilis* PB6 (6.6 × 10^9^ CFU/g), 1.0 Kg/MT feed, and (4) treatment-4 (T4): birds were fed a basal diet plus 4% Flavomycin, 0.3 Kg/MT feed.

A starter diet (crumble feed) from day 1 to 14, a grower diet (pellet feed) from day 15 to 28 including a finisher diet (pellet feed) from day 29 to 35 were provided depending upon age of the birds. In this trial, each replicate was housed in a clean and disinfected floor pen (15 m^2^/125 birds) with rice husk litter before the first day. The birds were fed on *ad libitum* basis with 24 h, 23 h, 20 h, 16 h and 12h artificial lighting conditions were maintained at the age of 1-3 days, 4-7 days, 8-14 days (second week), 15-21 days (third week) and onwards, respectively. Temperature was adjusted on age of the birds, that included 32°C in the first week with 200 watt tungsten light bulb, 30°C in the second week, 28°C in the third week, and then 25°C to the end of the study with artificial LED lights (30 watt) as described earlier (Cao et al., 2013). Since the study was conducted during hotter summer season, no artificial lighting was needed during day-time considering environmental temperature. The birds were immunized against Newcastle disease (ND) and Infectious bursal disease (IBD) at 5 and 9 days of age and booster/second dose at 22 and 17 days of age, respectively.

### 2.2 Feed formulation and preparation

The basal diet was prepared as per standard method (National Research Council, 1994) and was used as feed during the trial in 3 stages: day 1 – 14, day 15 – 28 and day 21 – 35 (Table 1). The feed ingredients (raw materials) were collected and weighed separately and blended properly with all feed additives (vitamins, minerals etc.) from a commercial feed miller. The probiotics and antibiotics were added in powder form in the basal ration according to the experimental design. Moisture, crude protein, crude fat, crude fiber and ash of the trial feed were analyzed for proximate analysis of feed, and however, amount of calcium and phosphorus and soluble chloride were analyzed as a part of mineral analysis.

**Table 1:** Ingredients and composition of the basal feed in broiler starter, grower and finisher ration (as feed basis, %).

Ingredients (%)	Starter(1 to 14 day)	Grower(15 to 28 day)	Finisher(29 to 35 day)
Maize	47.40	45.00	49.00
Rice polish- grade A	2.50	2.28	6.60
Wheat Flour Feed grade	7.00	7.00	10.00
Broken Rice	-	5.00	5.00
Soybean full fat (extruded)	7.00	8.00	3.00
Soybean meal 46% CP	23.5	19.30	16.00
Protein Concentration	4.00	4.00	3.00
Oil	2.00	3.00	3.00
Rape seed Meal 36%	3.00	3.00	1.00
Limestone	1.95	1.04	1.04
DCP^1^	0.50	0.50	0.50
Methionine MHA	0.20	0.32	0.32
Lysine-L	0.20	0.25	0.23
Vitamin and mineral premix 2	0.20	0.20	0.20
Enzyme	0.05	0.05	0.05
Salmonella killer^3^	0.10	0.15	0.15
Toxin (UTPP Biotect) 4	0.10	0.15	0.15
Salt	0.30	0.25	0.25
Sodium Bicarbonate	0.01	0.10	0.10
Choline^5^	**-**	0.08	0.08
Pro-plus^6^	**-**	0.03	0.03
Betaine^7^	**-**	0.05	0.05
Phyzyme 10000 FTU^8^	**-**	0.005	0.005
Mycocurb^9^	**-**	0.10	0.10
Quixalud^10^	**-**	0.05	0.05
Safe Guard^11^	**-**	0.05	0.05
Lysoforte^12^	**-**	0.05	0.05
**Total**	**100.00**	**100.00**	**100.00**
Calculated analysis metabolizable energy(Kcal/kg)	3039.82	3091.65	3095.8
Moisture	10.82	10.95	10.69
CP^13^	22.16	21.01	19.61
CF^14^	3.08	3.21	3.14
Ca	1.04	0.98	0.90
P	0.73	0.74	0.73

1 Vitamin and mineral premix provided the following nutrients in per kilogram of content: vitamin A, 600,000 IU; vitamin D_3_, 300,000 IU; vitamin E, 300 mg; vitamin B_2_, 600 mg; vitamin B_6_, 60 mg; vitamin B_12_, 4 mg; vitamin K_3_, 40 mg; nicotinic acid, 440 mg; folic acid, 100 mg; Zn, 14,400 mg; Cu, 7,200 mg; Mn, 14,400 mg; Se, 108 mg; essential amino acid, 64,800 mg.

2 DCP = Dicalcium Phosphate (a source of Ca)

3 Salmonella killer = feed additives which protects birds from *Salmonella* colonization and invasion

4 Toxin (UTPP Biotect) = essential components used in broiler ration for prevention of toxin generated from mold/fungus

5 Choline = vitamin-like essential nutrient in poultry feed for maintaining cell structure and fat metabolism

6 Pro-plus = protein tonic in poultry feed

7 Betaine = improves production performance as dietary supplementation in broiler ration

8 Phyzyme 10000 FTU = concentrated phytase feed enzyme in poultry feed for enhance digestibility to reduce feed cost

9 Mycocurb = mold inhibitor in poultry feed

10 Quixalud = effective against bacterial, fungi, and protozoa for improve feed conversion

11 Safe Guard = used as treatment and control of adult *Ascaridia galli* in broiler chickens

12 Lysoforte = a nutritional emulsifier in poultry ration designed for enhance digestion and absorption of energy-rich feed ingredients

13 CP = crude protein

14 CF = crude fiber

### 2.3 Growth performance

The body weight of birds was measured individually at the beginning of the study and every 7 days interval up to 35 day. For measuring average body weight of each replicate for a particular age, birds were selected randomly. A total of 60 birds were weighted and recorded the total weight at day 0, 7, 14 and 21 from each replicate using a measuring scale. However, 30 birds were weighted at the day 28 and 35 from each replicate. Thus, the mean value of live weight gain was measured from each replicate at 0, 7, 14, 21, 28 and 35 day. The feed consumption of each replicate was recorded in hardcopies on daily basis by subtracting the weight of the residual feed from the total quantity of feed offered. Successively, cumulative feed consumption for 7 days was determined for each replicate. Average daily growth and feed intake, feed conversion ratio (FCR), and percentage of mortality were calculated following the procedure described earlier (Khatun et al., 2017) to evaluate the growth performance of broilers included under this study.

### 2.4 Sample collection and processing

Samples were collected from broiler birds at day 21 and 35 for bacteriological evaluations. Intestinal lesion scoring and gut histopathology were accomplished from collected intestinal samples on day 28. However, serum was obtained from the collected blood samples on the same day (day 28) to perform a hemagglutination inhibition (HI) test for the assessment of antibody titer against Newcastle disease in each group of birds. For bacteriological evaluations, 12 birds (3 birds/replicate) were randomly selected from each group on day 21 and 35. These birds were euthanized after recording live body weight for collection of samples following aseptic measures. About 5 cm length of jejunum and ileum with their contents were collected in sterile zipper bags with sterile scissors and forceps, and transferred to the laboratory maintaining cool chain. Intestinal samples (1 g) were homogenized with 9 mL of 0.1% peptone water using a mortar and pestle, and a 10-fold dilution of sample was obtained. Subsequently, serial dilutions of each of the samples were accomplished using 0.1% peptone water.

### 2.5 Intestinal morphology

Intestinal lesion scoring and histopathological examination were conducted on day 28 for the evaluation of intestinal morphology. The samples were collected from euthanized birds of randomly selected two birds of a replication on day 28 (32 birds in total) for intestinal lesion scoring as per standard methods (Dahiya, Hoehler, Wilkie, Van Kessel, & Drew, 2005). In this scoring, a scale from 0 to 4 was used, where 0= apparently normal with no lesion; 0.5 = severely congested serosa and blood engorgement in mesentery; 1 = thin and friable intestine with small red petechiae; 2 = focal necrotic lesions; 3 = 1 – 2 cm long patches of necrosis; and 4 = diffused necrosis.

In histopathological evaluation, samples were collected from one of the two same euthanized bird of each replication (16 birds in total). A portion of 2 cm longitudinal section of the jejunum from each bird was collected in 10 percent phosphate buffered formalin. Further, four percent (4%) freshly prepared paraformaldehyde was used for fixation of jejunum tissues followed by embedding in paraffin blocks and sliced into 5 µm, deparaffinized in xylene, rehydrated, and finally, stained with hematoxylin and eosin stain. The tissue sections were examined by light microscope (100X). The grading of histopathological changes was performed as per standard protocol (Gholamiandehkordi et al., 2007) with moderate modification. In brief, histological scores are mainly based on the degree of villi fusion, erosion of the villi tips with loss of villi cellular content, abnormalities in epithelial tissues including volume of proteinaceous substances in the lumen of broiler intestine.

### 2.6 Bacteriological examination

Serial diluted (10^−1^ – 10^−8^ dilution) 0.1 mL each sample was inoculated into different selective agar media for bacterial culture. In this assessment, total *Bacillus, Bacillus licheniformis, Bacillus subtilis, Escherichia coli, Salmonella* spp., and *Clostridium perfringens* were enumerated using different culture media, viz., Hichrome Bacillus agar, MacConkey agar, Xylose lysine deoxycholate (XLD) agar, and Tryptose sulphite cycloserine (TSC) agar (Hi-Media, Mumbai, India). Plates of Hichrome Bacillus agar were incubated at 37°C for 24 h for total *Bacillus, B. licheniformis* and *B. subtilis* enumeration. Yellowish green to green colonies and irregular or creeping yellow colonies surroundings with yellow hue were considered for total *B. subtilis* and *B. licheniformis* counts respectively (Němečková, Solichová, Roubal, Uhrová, & Šviráková, 2011). In contrast, MacConkey agar, XLD agar culture media were used for *E. coli* and *Salmonella* respectively, and incubated at 37 °C for 24 h. However, TSC agar was used for *C. perfringens,* and incubated anaerobically using at 37°C for 24 h. Concentration of *C. perfringens* was tested on Tryptose-Sulfite-Cycloserine (TSC) agar, combined with perfringens (TSC) selective supplement (HiMedia, Mumbai, India) and 30% egg yolk emulsion following pour plate technique (Olnood, Beski, Choct, & Iji, 2015). After incubation of the agar plate, colonies were counted. Cultured plate containing 30 to 300 distinct colonies were counted and the number of colonies was then multiplied by the dilution factor to get respective bacterial counts. Thus, the result obtained was presented in log_10_ colony-forming units per gram of intestinal contents.

### 2.7 Immune responses

Twelve birds from each group (3 birds/replicate) were selected randomly and 0.1 mL of 0.5% sheep red blood cell (SRBC) was injected in brachial vein for immune response to SRBC on day 30. The immune response was measured after 5 days of inoculation by the micro titer haemagglutination method (Khaksefidi & Ghoorchi, 2006). Similar number of birds were included for the detection of serum antibody titers against NDV by hemagglutination inhibition (HI) test on day 28. The HI titer was expressed in log_2_ reciprocal of the highest serum dilution resulting in complete inhibition of hemagglutination activity.

### 2.8 Statistical analyses

Data for all parameters were analyzed for statistical significance using the Statistical Package for the Social Sciences (SPSS) version 23.0, IBM^Ⓡ^ Co., USA. Variance among the treatment groups was measured using the one-way ANOVA. The significant differences among the treatment groups were further determined by the Tukey honestly significant difference (HSD) test under significant result of ANOVA.

## 3 Results

### 3.1 Body growth performance

Live weight gain (BWG), average daily weight gain (ADWG), average daily feed intake (ADFI), and feed conversion ratio (FCR) were monitored weekly basis during the entire trial period (5 weeks). The live weight of trial birds at different ages are presented in Table 2. The BWG of T2 and T3 at the age of day 21 and 35 was found significantly higher (*p <* 0.05). However, lower BWG was observed in T1 and T4 at the age of day 21 and 35 (*p <* 0.05). The highest BWG was observed in T2 at the age of day 21 – 35 (Table 2).

**Table 2:** The effects of dietary treatments on growth performance of broilers at day 0, 7, 21, 28, 35 in four trial groups.^1^

Parameters^2^	Treatment				SEM^3^	*p*-value
	T1	T2	T3	T4		
BWG (g/chick)						
0 d	45.29 ± 0.11^a^	45.33 ± 0.08^a^	45.46 ± 0.12^a^	45.20 ± 0.08^a^	0.05	0.37
7 d	204.92± 0.50^a^	206.41 ± 0.46^a^	205.75 ± 0.78^a^	204.00 ± 0.59^a^	0.35	0.068
14 d	535.94 ± 3.93^a^	549.89 ± 2.67^a^	545.57 ± 3.15^a^	546.86 ± 3.36^a^	2.00	0.057
21 d	974.00 ± 3.34^a^	993.24 ± 2.97^b^	985.75 ± 3.68^ab^	984.89 ± 5.60^ab^	2.52	0.038*
28 d	1606.55 ± 16.98^a^	1658.11 ± 9.77^a^	1644.11 ± 11.86^a^	1637.62 ± 11.48^a^	7.52	0.079
35 d	2176.16 ± 14.57^a^	2258.17 ± 7.02^b^	2236.21 ± 10.49^b^	2231.39 ± 13.82^b^	9.43	0.003*
ADWG (g/bird/day)						
0-7 d	22.80 ± 0.08^a^	23.01 ± 0.08^a^	22.90 ± 0.13^a^	22.69 ± 0.09^a^	0.05	0.162
8-14 d	47.29 ± 0.6^a^	49.07 ± 0.32^a^	48.55 ± 0.52^a^	48.98 ± 0.45^a^	0.28	0.08
15-21 d	62.58 ± 0.25^a^	63.34 ± 0.59^a^	62.88 ± 0.92^a^	62.58 ± 0.77^a^	0.32	0.842
22-28 d	90.36 ± 2.58^a^	94.98 ± 1.16^a^	94.05 ± 1.97^a^	93.25 ± 1.83^a^	0.98	0.408
29-35 d	81.38 ± 4.08^a^	85.72 ± 1.75^a^	84.59 ± 2.42^a^	84.83 ± 1.04^a^	1.22	0.663
0-35 d	60.88 ± 0.41^a^	63.23 ± 0.20^b^	62.59 ± 0.30^b^	62.46 ± 0.39^b^	0.27	0.002*
ADFI (g/bird/day)						
0-7 d	25.40 ± 0.03^a^	25.34 ± 0.16^a^	25.20 ± 0.11^a^	25.20 ± 0.11^a^	0.06	0.514
8-14 d	64.21 ± 0.23^a^	64.70 ± 0.25^a^	65.09 ± 0.19^a^	65.23 ± 0.45^a^	0.17	0.124
15-21 d	93.12 ± 0.55^a^	93.50 ± 0.45^a^	93.06 ± 0.74^a^	93.35 ± 0.6^a^	0.27	0.946
22-28 d	131.58 ± 1.26^a^	130.29 ± 2.06^a^	134.63 ± 1.49^a^	133.37 ± 2.08^a^	0.89	0.358
29-35 d	147.31 ± 3.54^a^	142.72 ± 1.87^a^	138.91 ± 3.66^a^	145.32 ± 2.00^a^	1.53	0.247
0-35 d	95.18 ± 0.83^a^	93.05 ± 0.29^a^	94.10 ± 0.48^a^	94.10 ± 0.39^a^	0.31	0.098
FCR						
0-7 d	1.11 ± 0.006^a^	1.10 ± 0.005^a^	1.10 ± 0.008^a^	1.11 ± 0.006^a^	0.003	0.22
0-14 d	1.28 ± 0.009^a^	1.25 ± 0.004^a^	1.26 ± 0.009^a^	1.26 ± 0.005^a^	0.004	0.069
0-21 d	1.38 ± 0.003^a^	1.36 ± 0.007^b^	1.37 ± 0.002^ab^	1.37 ± 0.006^ab^	0.003	0.02*
0-28 d	1.42 ± 0.01^a^	1.37 ± 0.01^a^	1.37 ± 0.007^a^	1.40 ± 0.02^a^	0.01	0.179
0-35 d	1.55 ± 0.03^a^	1.47 ± 0.01^b^	1.49 ± 0.01^ab^	1.50 ± 0.01^ab^	0.01	0.021*
Mortality (%)						
0-35 d	4.60 ± 0.38^a^	3.40 ± 0.6^a^	4.00 ± 0.33^a^	3.60 ± 0.4^a^	0.23	0.278

a,b Within a row, means with different superscripts differ significantly (*p <* 0.05), one way ANOVA, Tukey's test).

1 The results are reported as means ± standard error.

⁎ Represents significant variation (*p <* 0.05) within a row.

2 BWG = Body Weight Gain; ADWG = Average Daily Weight Gain; ADFI = Average Daily Feed Intake; FCR = Feed Conversion Ratio; g = Gram; d = day.

3 SEM= Standard error of mean

The average daily weight gain (ADWG) during 0 – 35 day varied significantly among the four trial groups (*p <* 0.05) with a highest ADWG was observed in T2 (Table 2). However, T3 and T4 groups obtained nearly a similar ADWG, though T1 was found to have lowest body weight (Table 2).

Average daily feed intake (ADFI) among the different treatment groups with a 35 days trial period was found non-significant (*p>*0.05). However, a lower ADFI was observed in T2 and T3 (probiotic-supplemented groups) at 5^th^ week (Table 2). Additionally, during a 35 days trial period, the lowest ADFI was recorded in T2 (supplemented with *Bacillus licheniformis* DSM17236).

The impact of different dietary treatments on FCR of broiler poultry was found significantly varied (*p <* 0.05) at day 21 and 35 (Table 2). In this evaluation, a 35 days trial-period, T1 was found to be the highest FCR (1.55) than the other groups [T2 (1.47), T3 (1.49) and T4 (1.50)]. A lower FCR was documented in probiotic treated groups (T2 and T3) in each week of trial period where the birds were fed with *Bacillus licheniformis* DSM17236 (T2) and the FCR was found to be satisfactory.

A total of 78 broiler birds died in four trial groups during the entire study period where the highest mortality was recorded in T1 (n = 23) and the lowest in T2 (n = 17) trail group. However, second-highest mortality was observed in T3 (n = 20) (supplemented with *Bacillus subtilis* PB6). However, the mortality rate in broiler birds among different trial groups was found to be non-significant (Table 2).

### 3.2 Gut histopathology

**Fig. 1:**
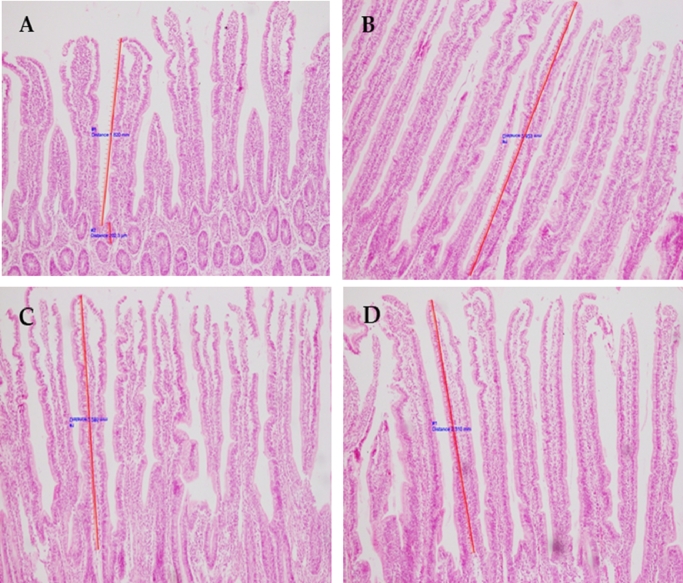
Cross sectional view of small intestine (jejunal villus) of broiler birds at 28 day of age (H & E X 100). (A): Moderate erosion of the villi tips with loss of villi material and flatten of the upper portion of the villi (T1); (B) Mild erosion with some separation of the tips and separation of the cells from the basement membrane (T2); (C) Moderate erosion of the villi tips with loss of villi material and flatten of the upper portion of the villi (T3), and (D) Moderate erosion of the villi tips with loss of villi material and flatten of the upper portion of the villi (T4).

Histopathology findings of small intestine (jejunum) tissue samples of all trial groups are presented in Table 3 and Fig. 1. A moderate erosion in the tips of the villi with loss of villi materials including flattening of the upper portion of the villi were observed in histopathology section of birds in T1, T3 and T4 trial groups. However, histopathology section of T2 group birds was found to be marginally better villi, slight erosion with some separation of the villi tips. The gut histomorphology was found to be relatively better in T2 group than the others. However, the histopathology scoring was found to be non-significant among the trial groups (Table 3).

**Table 3:** Comparison of histopathology, intestinal lesion scoring and immune response of broiler birds in four trial groups.^1^

Parameters	Treatment				SEM^2^	*p*-value
	T1	T2	T3	T4		
Gut histopathology (n = 4)	2.00 ± 0.00	1.00 ± 0.00	2.00 ± 0.00	2.00 ± 0.00	0.11	-
Intestinal lesion scoring (n = 8)	2.88 ± 0.13^a^	2.25 ± 0.25^a^	2.75 ± 0.14^a^	2.88± 0.24^a^	0.11	0.13
Antibody titer (log_2_) to SRBC antigen (n = 12)	2.67 ± 0.76^a^	3.17 ± 0.56^a^	2.75 ± 0.62^a^	2.83± 0.79^a^	0.33	0.959
Antibody titer (log_2_) against Newcastle virus (n = 12)	4.31 ± 0.50^a^	4.44 ± 0.48^a^	4.25 ± 0.25^a^	4.31± 0.34^a^	0.18	0.99

1 Values are mean and standard error. Mean values in the same row with different letters differ significantly (*p<*0.05). n = Number of birds of a treatment group examined for each test. ^2^ SEM = Standard error of mean

### 3.3 Intestinal lesion scoring

The birds from T1 and T4 had a greater average lesion score (2.88) than T2 and T3. However, average intestinal lesion score was found to be deviated marginally among two probiotic-supplemented groups (T2 and T3). The lowest intestinal lesion score was recorded in birds of T2 (*Bacillus licheniformis* probiotic supplemented group) with an average lesion score of 2.25. However, no significant difference (*p >* 0.05) of intestinal lesion scores among different treatment groups was found at the age of 28 day (Table 3).

### 3.4 Enumeration of intestinal bacteria

The intestinal (jejunal and ileal) microflora composition of broilers was examined at day 21 and 35 of age (Table 4). Unrelatedly to the trial groups, significant differences (*p<*0.05) were observed on the microbial populations at day 21 of age in jejunum samples for total *Bacillus* count (TBC) and day 35 of age for total *Bacillus* count (TBC), total *E. coli* count (TEC), total *Bacillus licheniformis* count (TBlC) and total *Bacillus subtilis* count (TBsC). The concentration of *Clostridium perfringens, Escherichia coli, Salmonella* was found to be decreased with age, at the same time, the colonization of both *Bacillus licheniformis* and *Bacillus subtilis* was elevated in both jejunum and ileum samples. In most cases, microbial count was presented higher in the ileum than jejunum samples at day 21 and 35.

**Table 4:** Comparison of bacterial enumeration (Log_10_ CFU/g) in intestinal (jejunum and ileum) content of birds with different treatments on day 21 and 35.^1^

Sample source	Differentbacterial count	Treatment				SEM ^2^	*p*-value
		T1(n = 12)	T2(n = 12)	T3(n = 12)	T4(n = 12)		
**Jejunum**	**21 day**						
	TCpC	2.63 ± 0.52^a^	1.53 ± 0.63^a^	2.47 ± 0.12^a^	2.43 ± 0.67^a^	0.26	0.482
	TSC	2.46 ± 0.67^a^	2.05 ± 0.58^a^	1.91 ± 0.84^a^	2.32 ± 0.99^a^	0.36	0.958
	TEC	7.36 ± 0.46^a^	5.35 ± 0.81^a^	5.53 ± 0.49^a^	7.07 ± 0.71^a^	0.37	0.096
	TBC	7.40 ± 0.13^a^	9.05 ± 0.07^b^	8.42 ± 0.19^cb^	8.27 ± 0.23^cd^	0.17	<0.001*
	TBlC	7.41 ± 0.71^a^	8.03 ± 0.20^a^	7.93 ± 0.71^a^	7.99 ± 0.21^a^	0.24	0.815
	TBsC	7.03 ± 0.29^a^	7.24 ± 0.90^a^	7.84 ± 0.15^a^	7.39 ± 0.28^a^	0.24	0.706
	**35 day**						
	TCpC	2.28 ± 0.70^a^	1.13 ± 0.05^a^	1.61 ± 0.35^a^	2.02 ± 0.91^a^	0.29	0.569
	TSC	1.92 ± 0.82^a^	2.18 ± 0.06^a^	1.76 ± 0.36^a^	2.60 ± 0.47^a^	0.24	0.676
	TEC	6.95 ± 0.25^a^	3.17 ± 0.70^b^	4.59 ± 0.38^ab^	5.23 ± 1.49^ab^	0.52	0.05*
	TBC	8.83 ± 0.15^a^	10.06 ± 0.20^b^	9.91 ± 0.19^cb^	9.20 ± 0.14^ac^	0.15	0.001*
	TBlC	8.79 ± 0.22^a^	9.41 ± 0.33^a^	8.89 ± 0.19^a^	8.82 ± 0.13^a^	0.12	0.232
	TBsC	8.80 ± 0.14^a^	9.27 ± 0.23^ab^	9.82 ± 0.20^b^	8.76 ± 0.20^a^	0.14	0.007*
**Ileum**	**21 day**						
	TCpC	2.72 ± 0.73^a^	1.92 ± 0.82^a^	2.59 ± 0.11^a^	2.64 ± 0.48^a^	0.28	0.77
	TSC	3.26 ± 0.35^a^	2.75 ± 0.30^a^	2.18 ± 0.80^a^	2.81 ± 0.23^a^	0.24	0.489
	TEC	7.10 ± 0.85^a^	5.69 ± 0.62^a^	5.44 ± 0.93^a^	6.19 ± 0.43^a^	0.37	0.422
	TBC	8.50 ± 0.05^a^	8.97 ± 0.07^a^	8.88 ± 0.15^a^	8.58 ± 0.17^a^	0.08	0.051
	TBlC	7.49 ± 0.73^a^	8.40 ± 0.21^a^	7.75 ± 0.68^a^	8.19 ± 0.11^a^	0.25	0.603
	TBsC	7.88 ± 0.13^a^	8.10 ± 0.16^a^	7.89 ± 0.80^a^	7.96 ± 0.18^a^	0.19	0.981
	**35 day**						
	TCpC	2.95 ± 0.74^a^	1.63 ± 0.98^a^	2.25 ± 0.44^a^	2.62 ± 1.17^a^	0.41	0.744
	TSC	2.95 ± 0.63^a^	1.04 ± 0.40^a^	2.03 ± 0.29^a^	2.77 ± 0.93^a^	0.34	0.167
	TEC	7.66 ± 0.07^a^	4.31 ± 0.67^b^	5.00 ± 0.48^b^	5.75 ± 0.56^ab^	0.39	0.003*
	TBC	8.67 ± 0.17^a^	10.32 ± 0.12^b^	10.21 ± 0.18^bc^	9.02 ± 0.12^a^	0.20	<0.001*
	TBlC	8.17 ± 0.17^a^	10.09 ± 0.10^b^	9.57 ± 0.20^bc^	8.96 ± 0.20^cd^	0.20	<0.001*
	TBsC	8.34 ± 0.02^a^	9.65 ± 0.15^b^	9.96 ± 0.18^bc^	8.86 ± 0.26^a^	0.18	<0.001*

1 Values of each row are mean and standard error of 12 birds of a trial group (n = Number of birds of a trial group examined for each test). Mean values in the same row with different letters differ significantly (*p <* 0.05). *Indicates significant variation (*p <* 0.05) within a row. TCpC = Total *Clostridium perfringens* count, TSC = Total *Salmonella* count, TEC = Total *E. coli* count, TBC = Total *Bacillus* count, TBlC = Total *Bacillus licheniformis* count, TBsC = Total *Bacillus subtilis* count. ^2^ SEM= Standard error of mean

### 3.5 Immune responses

Antibody response in different trial groups at day 35 has been presented in Table 3. Regarding antibody production against SRBC, no significant difference *(p > 0.05)* was found among different trial groups. However, antibody titer was found to be higher after 5 days of inoculation of SRBC in T2, T4 and T3 trial group respectively than the control group (T1). A similar pattern of immune response against NDV was observed in HI test. Result of HI antibody titer at day 28 among the different groups is shown in Table 3. Moderately higher antibody titer was documented in T2. However, no significant difference *(p > 0.05)* was observed in antibody titer to NDV among the groups.

## 4 Discussion

The poultry sector in Bangladesh has expanded rapidly since 1990s and this sector has an enormous contribution to the national economy regarding woman empowerment, income generation and supply of cheapest source animal protein for the country people (Joardar & Rahman, 2018). The commonly reported broiler diseases in Bangladesh are IBD, ND, Coccidiosis (Rahman et al., 2019), however, low prevalence of NE was reported in Bangladesh. Such disease distribution has confirmed based on the history, clinical findings including postmortem lesions (Hassan et al., 2016). This disease may be underreported or misdiagnosed with other broiler diseases in Bangladesh as the robust diagnostic tools are not being used. NE has a serious impact globally on poultry production causing severe economic losses due to reduced growth performance, increased mortality, huge treatment costs, and poor flock uniformity (Skinner, Bauer, Young, Pauling, & Wilson, 2010; Timbermont, Haesebrouck, Ducatelle, & Van Immerseel, 2011). The causal agent of NE is *C. perfringens*, a normal inhabitant in chicken intestinal microflora, usually found in low numbers in the posterior part of the gut. This pathogen cause disease through increase in number and toxins production in favorable condition (Jayaraman, Das, Saini, Roy, & Chatterjee, 2017; Timbermont, Haesebrouck, Ducatelle, & Van Immerseel, 2011). Therefore, appropriate management strategies should be included in the control approaches to curb the infection as an alternative to AGPs. Competitive exclusion by prebiotics, probiotics, enzymes and organic acids would be potential to support better gut health and to lessen incidence and severity of diseases, confirmed by several reports (Kaldhusdal, Schneitz, Hofshagen, & Skjerve, 2001; Zhou, Deng, Tao, Hu, & Hou, 2009).

In poultry production, this is very crucial to select safe and appropriate antibiotic alternatives that will give economic returns. Growth performances including BWG, ADWG, ADFI, and FCR are the major determinants used to assess the economic returns in broiler production (Zhang et al., 2021). In this study, BWG in T2 and T3 trial groups was found significantly higher at the age of day 21 and 35. Similarly, significant progress in ADWG of the birds during the entire production cycle (0-35 d) was observed as feed efficiency enhanced in probiotic supplemented trial groups (T2 and T3). Moreover, the feed conversion ratio (FCR) was found to be improved significantly (*p<*0.001) in probiotic treated groups. These findings are in agreement with several published reports where increased BWG and ADWG in probiotic supplemented feed (Deniz et al., 2011; Jayaraman et al., 2017; Kabir et al., 2005; Khatun et al., 2017; Lin et al., 2017; Zhang et al., 2021).

In this study, BWG was found to be significantly increased in the later weeks of a production cycle. This finding is corroborated by a study conducted in India as an improvement in growth performance was observed when probiotic microbial agent was supplemented to the finisher diet of broilers (Mohan, Kadirvel, Natarajan, & Bhaskaran, 1996). The beneficial effect of *B. licheniformis* and *B. subtilis* on growth performance including higher bone strength in broiler poultry (Mutuş et al., 2006)) due to the improved feed digestibility and rise of serum Ca and P by the action of beneficial bacteria in the gut (Rizzoli & Biver, 2020). The proper probiotic supplementation would render a suitable environment to assist microflora colonization in intestine and support to the better growth performance of birds (Jayaraman et al., 2017).

Moderate to mild erosion of the villi tips with loss of villi material and flatten of the upper portion of the villi were found in all trial groups, however, degree changes was relatively lesser in T2 trial group. This finding is sparsely supported by other research (Al-Baadani, Abudabos, Al-Mufarrej, & Alzawqari, 2016). However, in our study, there had no significant variation regarding gut histopathology and intestinal lesion scoring among the different groups. Moreover, T3 (*B. subtilis* PB6 supplemented group) demonstrated significant FCR along with an improved villi morphology. Nevertheless, a second highest mortality rate (4.00 ± 0.33%) with a poor immune response (against SRBC antigen and NDV) recorded in this trial group. This may be due to closely similar intestinal lesion score and gut histopathology score in T1, T3 and T4 groups. This signifies an imbalance condition of gut microflora including higher number of pathogenic bacteria especially *Clostridium perfringens* in the gut of broiler poultry. Moreover, birds were reared under low biosecurity farm this might enhance a higher colonization of pathogenic bacteria in the poultry gut causing nearly similar level of pathogenic lesions.

Higher antibody response in the probiotic groups (T2 and T3) than the control group was observed in this study could attribute to the *Bacillus* probiotics that enabling to assimilation of essential nutritional components for stimulating the immune production cells (Khaksefidi & Ghoorchi, 2006). A higher antibody titer was observed in the probiotic supplemented groups at 5 days of post-immunization is consistent with the finding of the present study (Khaksefidi & Ghoorchi, 2006). Further, the ability of probiotics to facilitate for production of serum and intestinal natural antibodies against several foreign antigens in chickens has been validated (Haghighi et al., 2005). The direct immunomodulatory impact of probiotics could stimulate the lymphatic tissue and the indirect impact through conservation of a balanced microflora in the intestine (Khaksefidi & Ghoorchi, 2006).

The results of present study relating to the intestinal microflora populations demonstrated that the dynamics of gut microorganism are affected by the feed supplementation of probiotics in broiler ration, and is responsible for significantly less growth of pathogenic bacteria, like *Clostridium perfringens, Escherichia coli* (Markowiak & Śliżewska, 2017). The concentration of *Clostridium perfringens, Escherichia coli, Salmonella* was found to be decreased with the age, at the same time, the colonization of both *Bacillus licheniformis* and *Bacillus subtilis* were found to be elevated in jejunum and ileum samples in probiotic treatment groups (T2 and T3). In most cases, microbial count was found higher in the ileum than jejunum samples at day 21 and 35. Nevertheless, a significant increase of other beneficial microorganisms like *Lactobacillus* spp. in the digestive tract of chickens was confirmed in several studies after inclusion of *Bacillus* probiotics in chicken feed (Whelan et al., 2019).

The main limitation of this study was that samples were taken from a fewer number of birds of trial groups that was not representative for histopathology, intestinal lesion scoring and immune response evaluations. Therefore, this makes insignificant result. Additionally, this could not be possible to establish a substantial association in all studied parameters of probiotic trial groups as the efficiency of a probiotic application depends on numerous factors, like type of strains, doses of probiotics, route of application, feed, and hygienic condition of the farm (Patterson & Burkholder, 2003). However, the study was conducted in broiler flocks with lower level of biosecurity measurements in hotter summer season. These might be the reasons for insignificant results in the stressful environments.

## 5 Conclusion

The study has demonstrated the impact of probiotics and AGP on the growth performance, gut morphology, intestinal microbiota and immune response in broiler chicken. Data of the present study confirmed that *Bacillus* based probiotics especially *Bacillus licheniformis* in broiler feed can support to enhance body weight gain, efficient feed conversion ratio including immune response in broiler chicken. Therefore, this probiotic could be used in broiler feed to facilitate cost effective broiler meat production and to minimize antimicrobial resistant associated with broiler rearing considering public health and food safety grounds.

## CRediT authorship contribution statement

**Mohammad Arif:** Formal analysis, Writing – original draft. **Md. Akteruzzaman:** Methodology. **:** Formal analysis. **Sk Shaheenur Islam:** Formal analysis, Writing – review & editing. **Bidhan Chandra Das:** Conceptualization, Methodology. **Mahbubul Pratik Siddique:** Writing – review & editing. **S. M. Lutful Kabir:** Conceptualization, Funding acquisition, Supervision, Writing – review & editing.

## Declaration of Competing Interest

The authors declare no conflict of interest. The funders had no role in study design, data collection and analysis, decision to publish, or preparation of the manuscript.
